# Tuberculous meningitis: where to from here?

**DOI:** 10.1097/QCO.0000000000000648

**Published:** 2020-04-30

**Authors:** Joseph Donovan, Guy E. Thwaites, Julie Huynh

**Affiliations:** a Oxford University Clinical Research Unit, Centre for Tropical Medicine, Ho Chi Minh City, Vietnam; b Nuffield Department of Medicine, Centre for Tropical Medicine and Global Health, Oxford University, Oxford, United Kingdom

**Keywords:** biomarkers, clinical trials, diagnosis, management, neurocritical, tuberculous meningitis

## Abstract

Tuberculous meningitis (TBM) is associated with significant mortality and morbidity yet is difficult to diagnose and treat. We reviewed original research published in the last 2 years, since 1 January 2018, which we considered to have a major impact in advancing diagnosis, treatment and understanding of the pathophysiology of TBM meningitis in children and adults.

**Recent findings:**

Studies have sought to identify a high sensitivity diagnostic test for TBM, with new data on modified Ziehl--Neelsen staining, urinary and cerebrospinal fluid (CSF) lipoarabinomannan and GeneXpert Ultra. Recent studies on CSF biomarkers provide a better understanding of the detrimental inflammatory cascade and neuromarkers of brain damage and suggest potential for novel host-directed therapy. Tryptophan metabolism appears to affect outcome and requires further study. Increased clinical trials activity in TBM focuses on optimizing antituberculosis drug regimens and adjuvant therapy; however, there are few planned paediatric trials.

**Summary:**

Tuberculous meningitis still kills or disables around half of sufferers. Although some progress has been made, there remains a need for more sensitive diagnostic tests, better drug therapy, improved management of complications and understanding of host-directed therapy if outcomes are to improve.

## INTRODUCTION

In 2018, tuberculosis (TB) affected 10 million people worldwide and killed 1.5 million [1]. The most fatal form of TB, tuberculous meningitis (TBM), occurs in 1–5% of those with TB. The highest risk populations are children under 5 years of age, the HIV co-infected, and immunocompromised. The disease burden in children may be increasing: a recent retrospective study from a high TB burden setting showing an alarming increase of TBM hospital admissions following global shortages of BCG (Bacille Calmette--Guerin) vaccine [2].

Despite advances in anti-TB chemotherapy, mortality from TBM remains unacceptably high (adults 50%, children 20%) [3,4]. Mortality in children is lower than in adults, but their developing brains render them at risk of unique age-specific neurological sequelae. Early diagnosis and timely initiation of appropriate therapy predict good outcome. But questions remain: ‘how can we diagnose TBM?’ and ‘what is appropriate therapy?’ Judicious neurocritical care of raised intracranial pressure (ICP) and blood pressure protect the brain from further damage, yet evidence guiding management is lacking. There have been recent efforts to improve understanding of pathophysiology with novel studies on biomarker signatures in TBM [5,6].

We searched PubMed for the term ‘tuberculous meningitis’ and found 328 publications between 1 January 2018 and the 7 December 2019. Here we provide an update on the diagnostics, pathophysiology, prognostics and management of TBM in adults and children. We highlight the current gaps in knowledge and the future trajectory of TBM clinical research. 

**Box 1:**
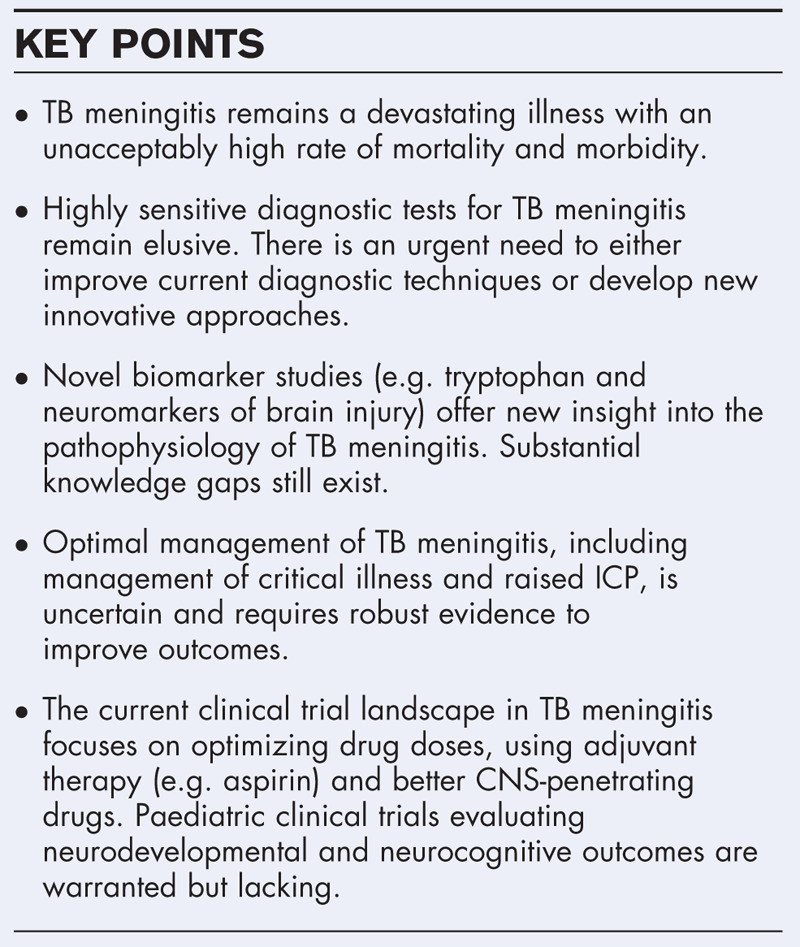
no caption available

## DIAGNOSTICS

Low bacterial numbers in cerebrospinal fluid (CSF) lead to challenges in *Mycobacterium tuberculosis* (Mtb) detection and diagnostic confirmation of TBM, particularly in children in whom large CSF volumes can be difficult to obtain. Current tests remain insufficiently sensitive to rule out TBM when they are negative. Comparing diagnostic test performance is confounded by the lack of a single gold standard reference test for TBM. As a result, use of the uniform case definition for clinical research [7] for test comparison is now commonplace.

New TBM diagnostics frequently seek to improve upon older tests. Ziehl--Neelsen smear microscopy of CSF for acid-fast bacilli is cheap and widely available, yet often insensitive unless performed by experienced microscopists using large volumes of CSF centrifuged at high speeds to concentrate Mtb. In Vietnam, South Africa and Indonesia, 618 individuals were enrolled into a prospective comparison of conventional Ziehl--Neelsen smear, modified Ziehl--Neelsen smear (using cytospin and permeabilization), GeneXpert MTB/RIF (Xpert) and mycobacterial culture [8^▪^]. Against a reference standard of definite, probable and possible TBM [7], sensitivities of conventional Ziehl--Neelsen, modified Ziehl--Neelsen, Xpert and mycobacterial culture were 33.9, 34.5, 25.1 and 31.8%, respectively [8^▪^]. Ziehl--Neelsen smear modifications did not improve diagnostic sensitivity.

The lipoarabinomannan (LAM) antigen is found in the cell wall of Mtb, and its detection represents an alternative to conventional TBM diagnostics. A prospective study of 550 adults (86% HIV co-infected) with suspected TBM in Zambia compared the diagnostic accuracies of CSF LAM and urinary LAM [Alere Determine TB LAM Ag assay (AlereLAM), Abbott, Chicago, Illinois, USA], against a reference standard of positive CSF mycobacterial culture [9^▪^]. Diagnostic sensitivities of CSF and urinary LAM were 21.9 and 24.1% respectively, suggesting, at least in this setting that these tests lack the sensitivity required for TBM diagnosis. In a subsequent study of 59 HIV co-infected individuals with suspected TBM in Uganda, lumbar CSF TB AlereLAM was compared against reference standards of positive GeneXpert MTB/RIF Ultra (Ultra), and definite plus probable TBM [10]. Whilst highly specific against these standards (96 and 95%, respectively), sensitivities of TB LAM were poor at 33 and 24%, respectively. However, a novel urine-based LAM assay with high-affinity Mtb specific antibodies and a silver-amplification step [Fujifilm SILVAMP, TB LAM (FujiLAM), Fujifilm, Tokyo, Japan] has a detection limit 30 times lower than AlereLAM [11]. In hospitalized adults with HIV co-infection, the FujiLAM had 35% higher sensitivity for TB diagnosis, and comparable specificity, to the AlereLAM [12^▪^]. Importantly, FujiLAM has yet to be assessed for the diagnosis of suspected TBM, HIV-uninfected or paediatric populations.

Xpert, recently superseded by Xpert Ultra [13], represents perhaps the most valuable diagnostic test for TBM currently available. Xpert Ultra are rapid PCR-based tests, crucially identifying rifampicin resistance when positive, guiding early therapy. Xpert Ultra is now a well established part of TBM testing. Ultra contains a larger reaction chamber than Xpert Ultra, with two additional different multicopy amplification targets [14]. Ultra has diagnostic superiority over Xpert in pulmonary TB, especially in samples expected to be paucibacillary [15]. Rapid endorsement by WHO [13] of Xpert Ultra came in 2017 following studies demonstrating its improved sensitivity in TB diagnosis [16^▪^]. An initial study suggested diagnostic superiority of Ultra over Xpert in 23 HIV co-infected adults with TBM [16^▪^]. Ultra and Xpert sensitivities for Mtb detection in CSF were 70 and 43%, respectively, against a reference standard of definite and probable TBM. Subsequent small studies have further assessed Ultra and Xpert for extrapulmonary TB diagnosis [17–19]. In 43 HIV uninfected adults with suspected TBM in China, Ultra had a higher sensitivity for Mtb detection in CSF than Xpert [19/43 (44.2%) vs. 8/43 (18.6%), respectively], against a reference standard of definite, probable and possible TBM. However, many confirmed cases in the Ultra group were confirmed by Ultra only. Paediatric data on the performance of Xpert Ultra in TBM is lacking. In a recent paediatric study of 28 children with definite, probable or possible TBM, diagnostic sensitivity of Xpert was 46.2% (6/13) against a total TBM diagnostic score of at least 10 points [20]. Large studies comparing Ultra and Xpert for TBM diagnosis in both adults and children are expected to be published soon.

## PATHOPHYSIOLOGY

**FIGURE 1:**
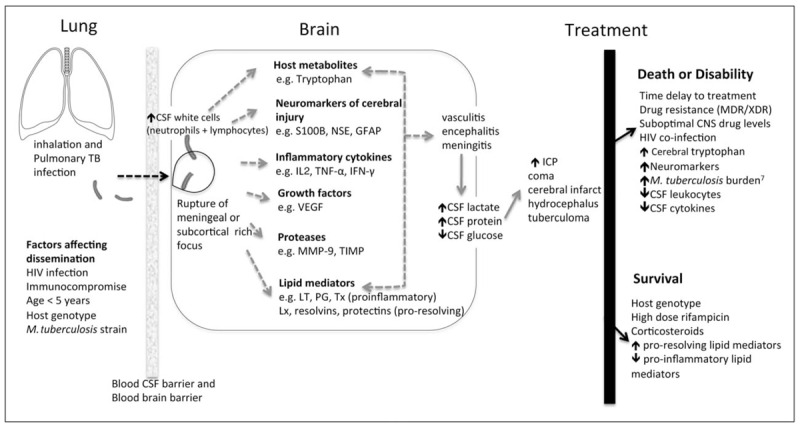
Overview of the pathophysiology of tuberculosis meningitis^∗^. Mortality of TBM is attributed to *Mycobacterium tuberculosis* and its interaction with the host immune response. Once *M. tuberculosis* enters the brain or meninges, a detrimental immunoinflammatory response including inflammatory cytokines, proteases, lipid mediators, neuromarkers and tryptophan metabolites, is triggered [1–6]. This leads to the cerebral disorder and complications known to occur in TBM. Knowledge gaps exist in the mechanism of bacterial invasion into the CNS and the underlying biologic pathways (dotted arrows) leading to the disease process. CSF, cerebrospinal fluid; GFAP, glial fibrillary acidic protein; HIV, human immunodeficiency virus; ICP, intracranial pressure; IFN, interferon; IL, interleukin; LT, leukotriene; Lx, lipoxins; MDR, multidrug-resistant; MMP, matrix metalloproteinases; NSE, neuron-specific enolase; PG, prostaglandin; TB, tuberculosis; TIMP, tissue inhibitor of matrix metalloproteinases; TNF, tumour necrosis factor; Tx, thromboxane; VEGF, vascular endothelial growth factor; XDR, extensively drug-resistant. Adapted from Thwaites and Tran [27]. Figure references [6,21,22^▪▪^,^23^–^27^].

Corticosteroids are a widely used adjunctive therapy in the treatment of TBM but how they improve outcome remains unknown. Recent insights into possible mechanisms driving the inflammatory response in TBM include the regulated balance of inflammatory eicosanoids and host protective mediators (specialized pro-resolving mediators). A study using a lipid-mediator profiling approach demonstrated a specialized pro-resolving mediator profile in CSF associated with disease severity and mortality in adults with TBM. Furthermore, the prothrombotic mediator thromboxane A2 was reduced in CSF in adults with TBM randomly assigned aspirin compared with those who received placebo [21] (Fig. 1).

A potential role for tryptophan metabolism in TBM outcome has recently been demonstrated. In an observational cohort study of the metabolomes of 33 HIV uninfected Indonesian adults with TBM, CSF tryptophan concentrations were lower in individuals who survived, compared with those who died (by nine times), and compared with controls (by 31 times) [22^▪▪^]. More than 10 genetic loci were identified as being predictive of CSF tryptophan concentrations in the TBM cohort suggesting the influence of host genotype on outcome.

Biomarker studies of cerebral injury in paediatric TBM have helped gain further insight. In a study of 44 children with TBM-associated hydrocephalus, 11 healthy controls and 9 children with pulmonary TB, neuromarkers of brain damage; S100B, neuron-specific enolase and glial fibrillary acidic protein, were elevated for at least 3 weeks in the TBM group [6]. Neuromarker concentrations increased over time and predicted poor outcome. Furthermore, high concentrations of neuromarkers and inflammatory markers were uniquely compartmentalized to the site of infection (ventricular CSF) but not identified in lumbar CSF or serum. A subsequent transcriptomic study demonstrated this distinct biomarker signature to be unique to TBM when compared with non-TBM controls and was associated with neuronal excitotoxicity [28^▪▪^]. For the first time, this offers insight into the possible mechanism of cerebral injury in TBM and potential impact on critical neurodevelopment of the immature brain in young children.

## PROGNOSIS

Prognostic models have been developed, which may aid clinicians in identifying patients at the greatest risk of death [29]. However, disease complications may change the likelihood of a poor outcome but are not captured by models, which use only baseline data. Using data from 1048 adults with TBM from three randomized controlled trials and one prospective observational study at two Vietnamese hospitals from 2004 to 2016, a dynamic prediction model was developed where follow-up measurements of Glasgow coma score (GCS) and plasma sodium were incorporated [30^▪^]. In HIV-uninfected and HIV co-infected individuals, higher GCS indicated lower mortality with hazard ratios of 0.76 (95% CI 0.71–0.81) and 0.85 (95% CI 0.81–0.91), respectively. In HIV-uninfected individuals, plasma sodium concentrations of 140 mmol/l; higher than normal for TBM, were associated with worse prognosis from baseline until day 10 of treatment than plasma sodium concentrations of 135 mmol/l. However, by day 30 these higher sodium concentrations were associated with improved survival. In HIV co-infected individuals, higher sodium concentrations associated with better prognosis across all prediction time points. No recent modelling studies predict prognosis in children.

## TUBERCULOUS MENINGITIS CRITICAL ILLNESS

The evidence base guiding supportive, medical and neurosurgical management of critically ill individuals with TBM is limited, with numerous research gaps recently highlighted [31]. TBM-associated hyponatraemia is common and may contribute to raised ICP. In a recent randomized trial in India, intravenous and oral salt with or without fludrocortisone was used in the treatment of 37 adults with TBM-associated hyponatraemia secondary to cerebral salt wasting. In the fludrocortisone group, correction of hyponatraemia was faster (4 vs. 15 days); however, severe hypokalaemia and hypertension developed in two patients requiring discontinuation of fludrocortisone in these cases [32^▪^].

In TBM, hydrocephalus, cerebral infarction, paradoxical reactions including tuberculomas, neurological immune reconstitution inflammatory syndrome in HIV co-infected individuals and seizures may all reduce GCS, a tool routinely used for patient assessment. The cause of seizures in TBM is multifactorial; data regarding the cause and timing of seizures in patients with TBM is scarce, and their incidence appears to vary substantially between populations [31]. A recent study of Indian adults with TBM described seizures in 34% (27/79) of patients, and abnormal electroencephalogram changes were observed in 85% (17/20) of patients who had seizures and an electroencephalogram performed [33].

Detection and monitoring of raised ICP, a common endpoint of TBM neurocomplications, is limited by global availability of gold standard invasive monitoring methods. Further evidence to support use of noninvasive ICP monitoring techniques, such as optic nerve sheath ultrasound, is needed. Optimal management of raised ICP is uncertain; previous neurosurgical intervention studies for TBM-associated hydrocephalus have largely focused on children. The permanent relief of raised ICP in TBM-associated obstructive hydrocephalus can be achieved through ventriculoperitoneal shunting or endoscopic third ventriculostomy (ETV), yet no study to date has shown one technique to be consistently superior to the other in TBM. In a recent study of TBM-associated hydrocephalus in India, children were randomized to ETV or ventriculoperitoneal shunting [34]. Success rates were 65% (17/26) and 61% (16/26), respectively. Poor outcomes for both procedures were linked to increased disease severity. A recent study assessed outcomes in individuals (predominantly adults) with TBM-associated hydrocephalus and HIV co-infection, either receiving (*n* = 15) or not receiving (*n* = 15), antiretroviral therapy (ART) [35^▪^]. In the ART and non-ART groups there were 4/15 (27%) and 10/15 (67%) deaths, respectively. Whilst this suggests outcomes after ventriculoperitoneal shunting for TBM-associated hydrocephalus are better once ART is commenced, CD4 counts in the ART group had generally recovered well. Outcomes in patients newly starting ART after HIV diagnosis at the time of TBM presentation are uncertain.

Given the complex and challenging nature of critical illness in TBM, patient assessment proformas and a priorities checklist have recently been developed [36] with the aim of standardizing clinical review and prioritizing likely causes of acute deterioration. However, these tools require validating.

## CLINICAL TRIALS

Whilst many questions regarding optimal TBM management remain unanswered, the current TBM research field is dynamic. A search of clinicaltrials.gov and ISRCTN trial registries identified 11 relevant prospective randomized studies or clinical trials in TBM (Table 1). Current trials in TBM can generally be separated into those aiming to optimize Mtb killing, and those using adjunctive therapies to prevent and manage disease complications. The uncertainty surrounding optimal anti-TB chemotherapy regimens and doses in TBM is underlined by the current trial panorama, where optimized anti-TB chemotherapy trials dominate. Given the poor central nervous system penetration of rifampicin, and its importance as a first line therapy, multiple studies of high-dose rifampicin, with or without linezolid, are underway or due to start. High-dose isoniazid therapy in rapid acetylators is also under investigation.

An evidence base is forming for adjunctive aspirin therapy in TBM. Aspirin may reduce the incidence and promote the resolution of TBM-associated brain infarcts, as shown in a recent phase 2 trial of adjunctive aspirin therapy in 120 HIV-uninfected adults with TBM in Vietnam [37]. Large phase 3 trials are required. In the adult LASER-TBM trial (NCT03927313), high-dose rifampicin and linezolid will be trialed with or without 1000 mg adjunctive aspirin for 8 weeks. In the SURE trial (ISRCTN40829906), children with TBM will be randomized to 6 or 12-month anti-TB chemotherapy, and will also undergo a second randomization to aspirin 20 mg/kg or placebo. Adjunctive dexamethasone is now commonly used in HIV-uninfected individuals with TBM. Whether all HIV-uninfected individuals with TBM should receive corticosteroids, and whether there is benefit in HIV co-infected individuals, is currently under investigation [38,39]. LAST ACT (NCT03100786) is the first study to personalize anti-inflammatory therapy in TBM by host leukotriene A4 hydrolase genotype.

Most therapeutic advances in TBM have occurred in adult studies, however, drug pharmacokinetics and TBM treatment outcomes are different in children. Children generally metabolize drugs differently; drug clearance being inversely proportional to age [40]. Although children suffer lower mortality than adults, other treatment outcomes, such as neurodevelopmental disabilities are paediatric-specific [40]. In addition to age-appropriate pharmacokinetic data, clinical trials, which characterize neurodevelopmental and cognitive outcomes in paediatric TBM, are needed to evaluate drug efficacy appropriately. Conducting such clinical trials in a real world setting has been challenging with recent data from the TBM-KIDS (NCT02958709) trial showing that only 21/3371 (<1%) children screened with clinical meningoencephalitis met enrolment [41]. Notably, this trial included children with confirmed and probable TBM; potentially excluding those with early-stage disease who met the definition of possible TBM. To date TBM-KIDS, an open label randomized trial in Indian and Malawian children evaluating efficacy of high-dose (30 mg/kg) oral rifampicin, and SURE, a factorial and noninferiority trial evaluating the efficacy of shortened intensified anti-TB chemotherapy and adjuvant aspirin, represent the only clinical trials in paediatric TBM.

## CONCLUSION

TBM is difficult to diagnose, optimal drug regimens are unknown, and management of common complications poorly evidence-based. The true global burden of TBM is hard to ascertain, and many cases likely remain undiagnosed [42]. Standardization of research methods and of study reporting, facilitated by global networking through the TBM International Research Consortium, first convened in 2009, represents progress. The consortium has also recently published a collection of reviews and opinion pieces on TBM (https://wellcomeopenresearch.org/collections/tbmeningitis). Recent and new clinical trials are encouraging, however, there is still a long way to go to convert an increasing evidence base into improved clinical outcomes. Few studies exist in paediatric TBM, despite the high burden of disease in this population. Studies of complications causing critical illness of TBM are particularly lacking. Research priorities include developing a high-sensitivity diagnostic test with the ability to exclude TBM. In addition, evidence to support shortened intensified anti-TB chemotherapy regimens would be welcome. Unfortunately, the current prognosis of TBM, particularly of severe cases, remains poor, and mortality and morbidity from this disease unacceptably high.

**Table 1:** Registered current and future trials in tuberculosis meningitis, listed by start date

Trial short title	Design	Study population	Intervention arms(s)	Countries recruiting	Sample size	Recruitment period	Primary outcome	Clinical trial registry number
TBM-KIDS	Randomised, open-label trial phase II	Age ≥6 months Age < 12 years Weight >6 kg Clinical TBM	Control: standard anti-TB therapy Interventions: High-dose rifampicin (30 mg/kg) containing regimen, OR high dose rifampicin (30 mg/kg) containing regimen plus substitution of ethambutol for levofloxacin (20 mg/kg)	India, Malawi	120	February 2017 -- ongoing	PK, functional, neurocognitive and safety endpoints	NCT02958709
ACT HIV	Randomised, double blind, placebo controlled phase III trial	Age ≥18 years Clinical TBM HIV co-infected	Control: standard of care without dexamethasone Intervention: adjunctive dexamethasone tapering regimen	Vietnam, Indonesia	520	June 2017 -- ongoing	Overall survival until 12 months	NCT03092817
RiFT	Randomised, open-label, phase II trial	Age ≥18 years Clinical TBM	Control: standard anti-TB therapy Interventions: regimens containing high dose (20 mg/kg) intravenous then oral 35 mg/kg rifampicin OR high dose (35 mg/kg) oral rifampicin	Uganda	60	September 2017 -- ongoing	PK parameters, composite safety endpoint	ISRCTN 42218549
LAST ACT	LTA4H genotype stratified, randomised (CC or CT genotypes), double blind, placebo-controlled phase III noninferiority trial	Age ≥18 years Clinical TBM HIV-uninfected	Control: standard of care without dexamethasone Intervention: adjunctive dexamethasone tapering regimen	Vietnam	720	February 2018 -- ongoing	All-cause mortality or new neurological event until 12 months	NCT03100786
Optimizing anti-TB therapy in adults with TBM	NAT2 stratified, randomised, parallel group trial	Age 18–65 years Clinical TBM	Control: standard anti-TB therapy in normal acetylators Interventions: rapid acetylators randomised to standard isoniazid containing regimen OR high dose isoniazid containing regimen	China	676	March 2019 -- ongoing	Number of participants with death or severe disability at 12 months	NCT03787940
LASER-TBM	Randomised, parallel group, multiarm phase IIa trial	Age ≥18 years Clinical TBM HIV co-infected	Control: standard anti-TB therapy Interventions: high-dose regimen containing 35 mg/kg oral rifampicin, and oral linezolid 1.2 g, OR high-dose regimen plus aspirin 1 g	South Africa	100	June 2019 -- ongoing	Treatment related adverse events	NCT03927313
SIMPLE	Randomised, open label phase II trial	Age ≥18 years Clinical TBM	control: anti-TB regimen containing 35 mg/kg rifampicin (high dose) Interventions: High-dose rifampicin regimen with oral linezolid 600 mg, OR high-dose rifampicin regimen with oral linezolid 1.2 g	Indonesia	36	June 2019 -- ongoing	Linezolid blood and CSF PK	NCT03537495
HARVEST	Randomised, double-blinded, placebo-controlled, phase III trial	Age ≥18 years Clinical TBM	Control: standard fixed dose anti-TB therapy Intervention: Regimen containing additional four oral rifampicin 300 mg capsules	Indonesia, South Africa, Uganda	500	Estimated recruitment start - January 2020	6-month survival	ISRCTN 15668391
SURE	Multifactorial randomiization (×2), open label phase III trial	Age >28 days Age <15 years Clinical TBM	Control: standard 12-month anti-TB therapy Interventions: 6-month intensified therapy Oral aspirin 20 mg/kg or placebo	India, Uganda, Vietnam, Zambia, Zimbabwe	400	Estimated recruitment start - February 2020	All-cause mortality at 48 weeks, neuro-development at 48 weeks	ISRCTN40829906
ALTER	Randomised, open-label, phase II trial	Age >18 years Clinical TBM	Four arms: standard dose rifampicin containing regimen with/without oral linezolid 1.2 g, or high-dose (35 mg/kg) rifampicin with/without oral linezolid 1.2 g	Uganda	60	Estimated recruitment start -- April 2020	CSF PK	NCT04021121
INTENSE- TBM	Randomised, placebo controlled (for aspirin), phase III, 2 × 2 factorial superiority trial	Age ≥15 years Clinical TBM	Four arms: standard anti-TB therapy OR high dose (35 mg/kg) oral rifampicin containing therapy and plus oral linezolid 1.2 g added to standard anti-TB therapy, with/without aspirin 200 mg	Ivory Coast Madagascar South Africa Uganda	768	Estimated recruitment start -- April 2020	Rate of all-cause death until 40 weeks	NCT04145258

CSF, cerebrospinal fluid; ISRCTN, prefix to unique clinical trials number in the International Standard Randomised Controlled Trial Number registry; LTA4H, leukotriene A4 hydrolase; NAT2, N-acetyltransferase type 2; NCT, prefix to unique clinical trials number on clinicaltrials.gov; PK, pharmacokinetic; TBM, tuberculous meningitis.

## Acknowledgements

None.

### Financial support and sponsorship

The authors are supported by the Wellcome Trust, United Kingdom.

### Conflicts of interest

There are no conflicts of interest.

## REFERENCES AND RECOMMENDED READING

Papers of particular interest, published within the annual period of review, have been highlighted as:

▪ of special interest
▪▪ of outstanding interest
